# Supplementary material for: Chronic activation of endothelial MAPK disrupts hematopoiesis via NFKB dependent inflammatory stress reversible by SCGF
Source: Nat Commun. 2020 Feb 3;11:666. doi: 10.1038/s41467-020-14478-8 (PMC6997369; doi:10.1038/s41467-020-14478-8)
Supplement: Supplementary file 1 — Supplemental Information [file 41467_2020_14478_MOESM1_ESM.pdf]

Supplemental Information

Chronic activation of endothelial MAPK disrupts hematopoiesis via NFKB dependent inflammatory stress reversible by SCGF

Ramalingam et al.

Supplemental Information

Supplemental Figure 1

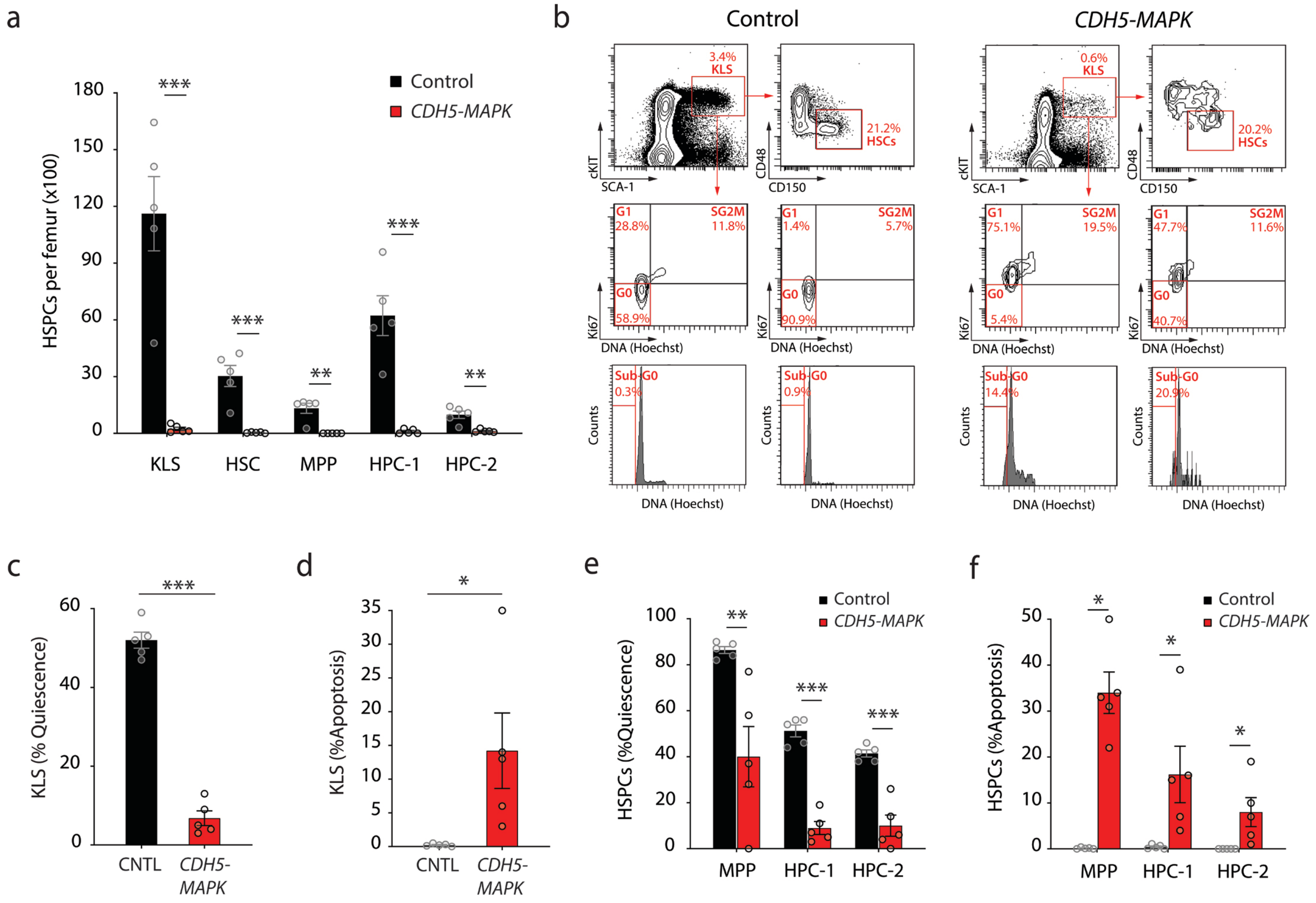

Supplemental Figure 1. HSPCs of *CDH5-MAPK* mice display loss of quiescence and increased apoptosis. a) Total number of phenotypic HSCs and HSPCs per femur assessed by Flow cytometry (n=5 mice/cohort). **b)** Representative contour plots demonstrating gating strategy to assess HSC cell cycle and apoptosis by Flow cytometry. **c-f)** KLS cells and HSPCs from *CDH5-MAPK* mice demonstrate loss of quiescence and increased apoptosis (n=5 mice/cohort). Error bars represent sample mean ± SEM. Statistical significance was determined using two-tailed unpaired Student's t-test. * P≤0.05; ** P<0.01; *** P<0.001; n.s. P>0.05.

Supplemental Figure 2

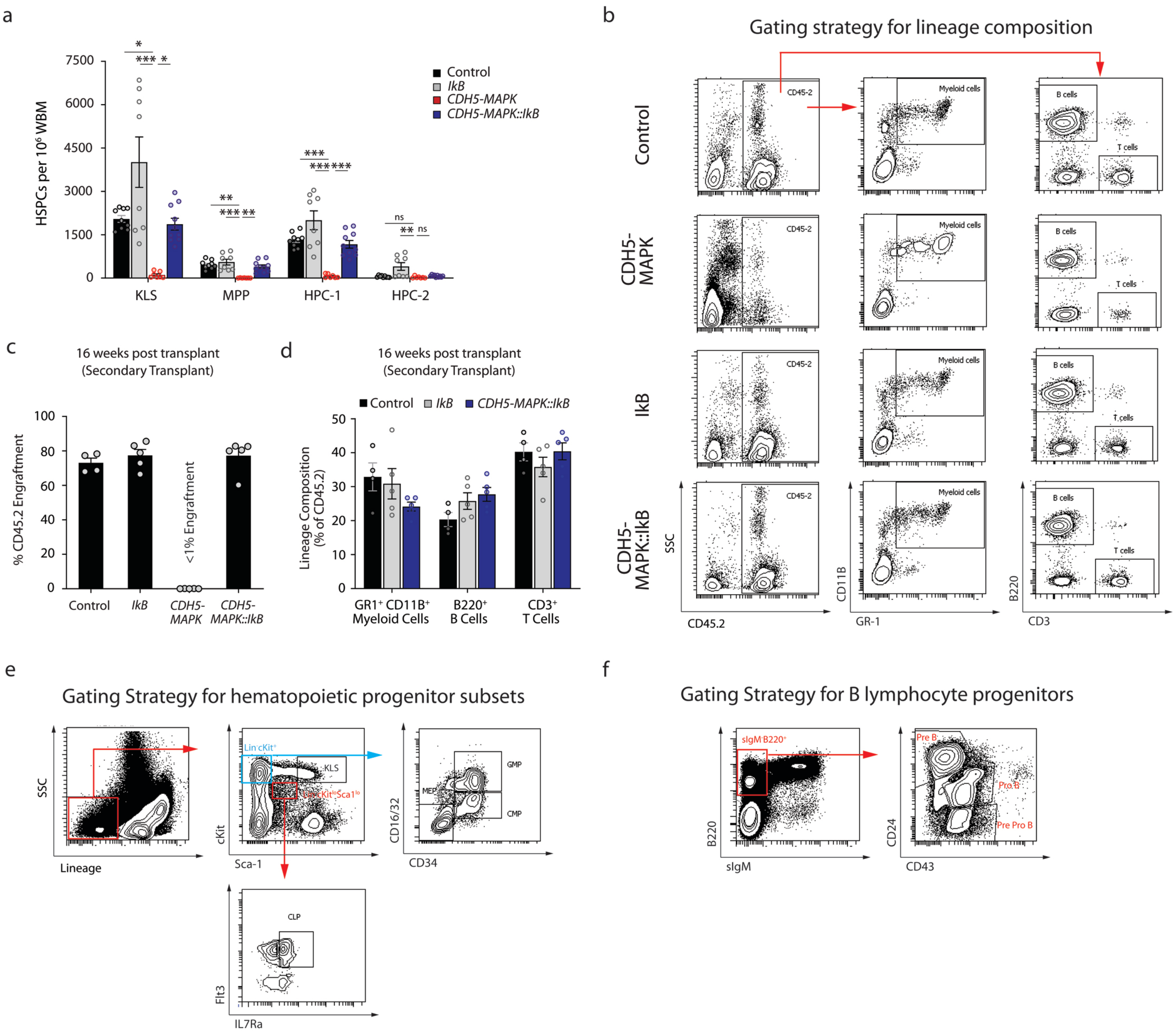

Supplemental Figure 2. Endothelial NF-κB inhibition restores hematopoietic defects in *CDH5-MAPK* mice. a) Frequency of phenotypic HSPCs per 10^6 femur cells assessed by Flow cytometry (n=7-10 mice/cohort). **b)** Representative contour plots demonstrating gating strategy for quantifying lineage composition of long-term engrafted CD45.2+ hematopoietic cells **c, d)** Secondary transplantation assay wherein WBM cells from long-term engrafted primary recipients were isolated and transplanted into pre-conditioned CD45.1 recipient mice (2x10^6 donor WBM cells per secondary recipient) . (n=4-5 recipients/cohort; n=5 donors per cohort). **e, f)** Representative contour plots demonstrating gating strategy for quantifying hematopoietic progenitors and B lymphocyte progenitors by Flow cytometry. Error bars represent sample mean ± SEM. One-way ANOVA for multiple comparisons and Tukey's correction was performed to determine significance. * P≤0.05; ** P<0.01; *** P<0.001; n.s. P>0.05.

Supplemental Figure 3

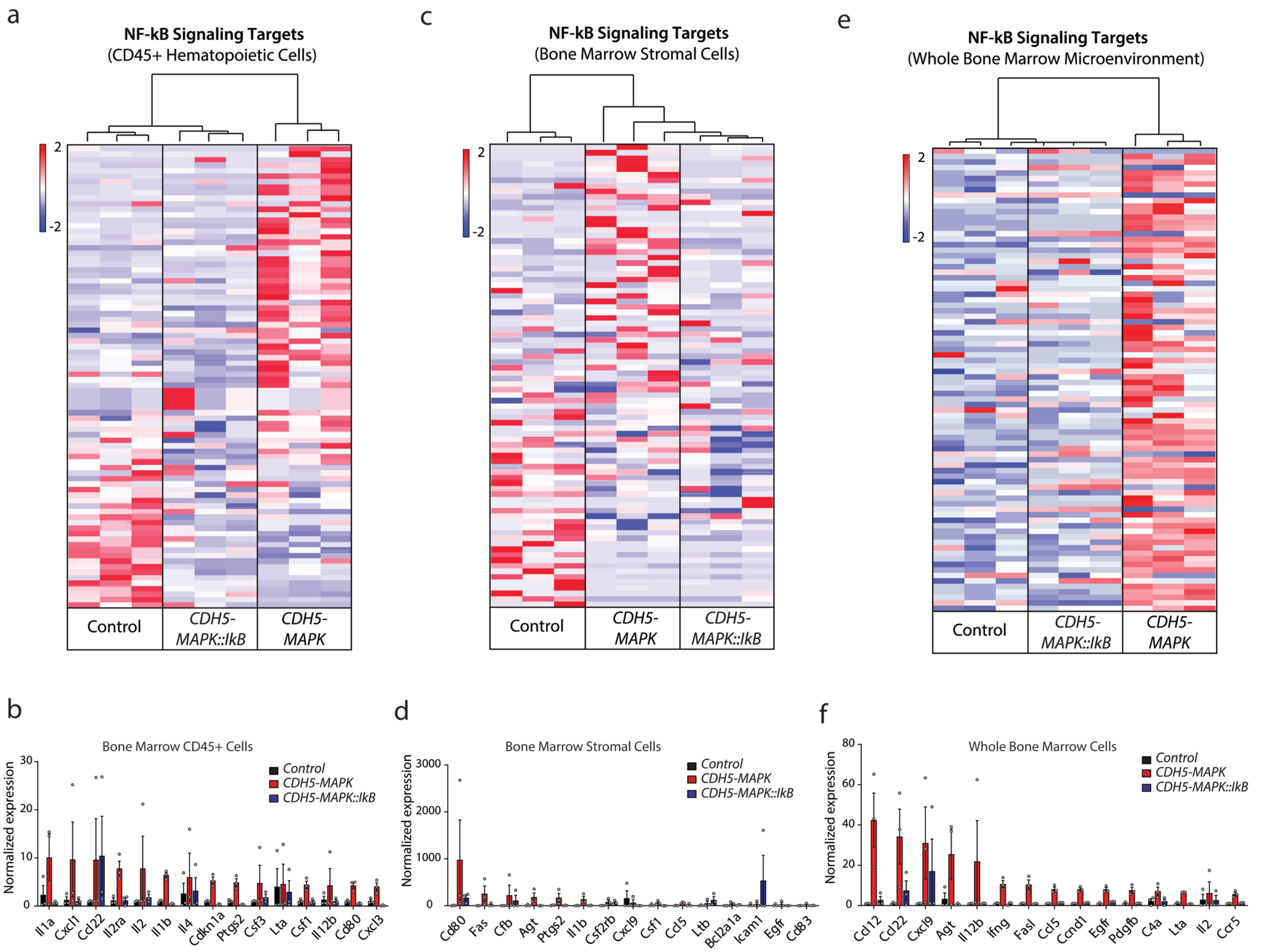

Supplemental Figure 3. Endothelial NF-κB inhibition suppresses BM inflammation in *CDH5-MAPK* mice. a-f) Heatmaps and bar graphs representing gene expression analysis (RT-qPCR arrays for NF-kB signaling targets) of FACS sorted cells including **a, b)** BM CD45+ hematopoietic cells, **c, d)** BM stromal cells, and **e, f)** unfractionated whole BM cells demonstrating that *CDH5-MAPK* mice display an increased expression of NF-kB regulated inflammatory genes. Importantly, crossing *CDH5-MAPK* with *Tie2.IkB-SS* mice (*CDH5-MAPK::IkB*) suppresses BM inflammation observed in *CDH5-MAPK* mice. Dendrograms represent unsupervised hierarchical clustering of the entire dataset. *Actb* used as reference gene for normalization (n=3 mice/cohort). Error bars represent sample mean ± SEM. Color scales represent normalized gene expression values. Raw data for Heatmaps included in Supplemental Table 2 and Source Data.

Supplmental Figure 4

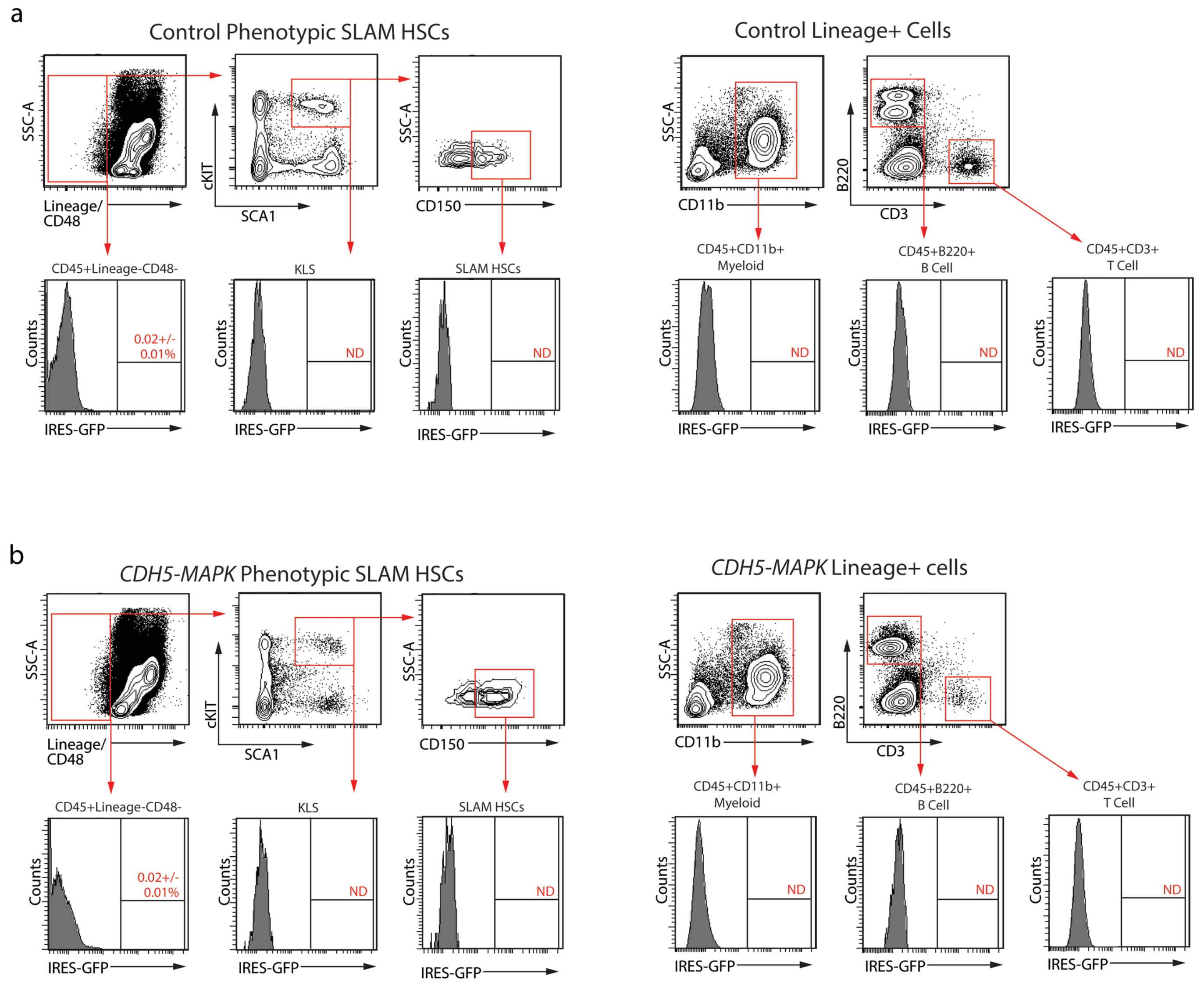

Supplemental Figure 4. ***CDH5-creERT2*** **is not expressed in HSCs or hematopoietic cells within BM of** ***CDH5-MAPK*** **mice. a, b)** Representative contour plots demonstrating gating strategy for assessment of *cre* mediated recombination and GFP expression within HSCs and mature hematopoietic cells of **a)** control and **b)** *CDH5-MAPK* mice. (n=3 mice per cohort).

Supplemental Figure 5

a

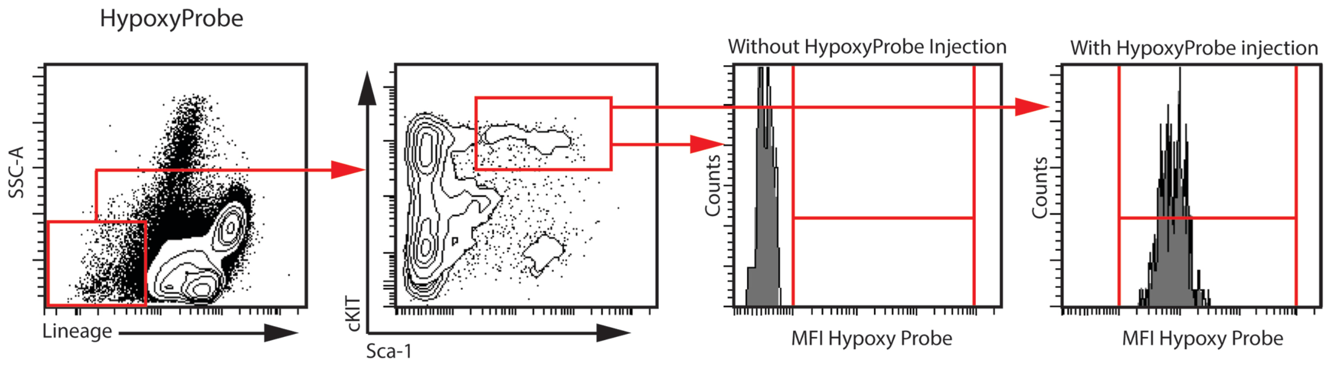

b

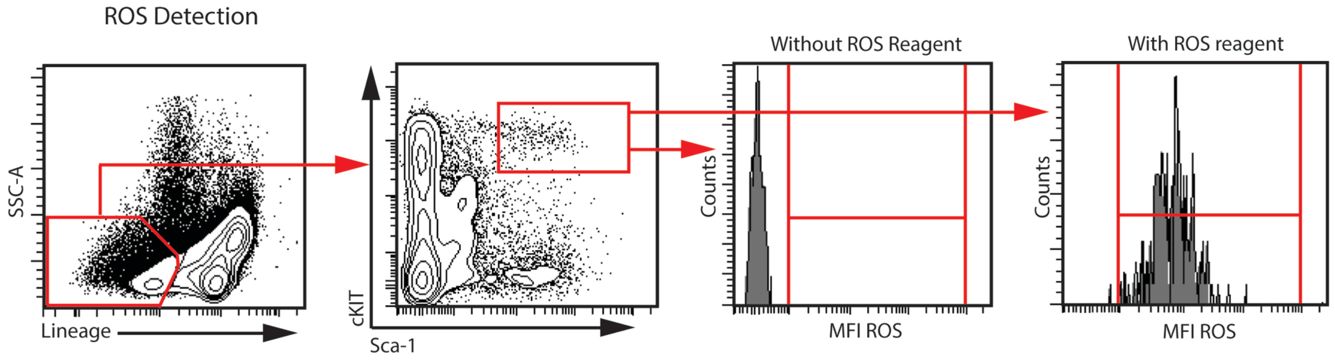

Supplemental Figure 5. Gating strategy for assessment of hypoxia and ROS. a) Representative contour plots demonstrating gating strategy for assessment of hypoxia in HSPCs by Flow cytometry. **b)** Representative contour plots demonstrating gating strategy for assessment of ROS in HSPCs by Flow cytometry. MFI denotes Mean Fluorescence Intensity. FMO controls were utilized to establish gates.

Supplemental Figure 6

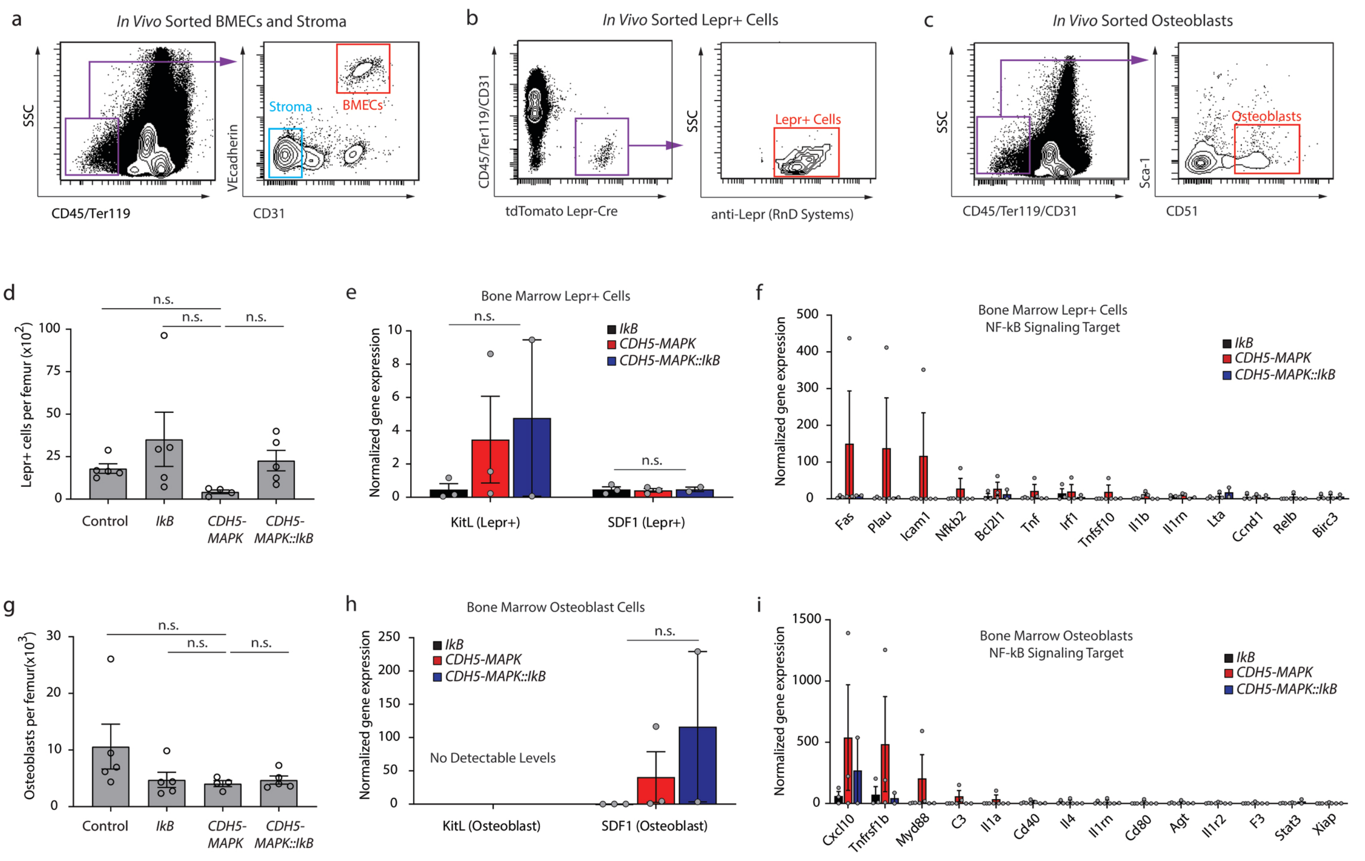

Supplemental Figure 6. Analysis of Lepr+ cells and osteoblasts within BM. a-c) Representative contour plots demonstrating gating strategy for analysis of the indicated cell types by Flow cytometry. The fidelity of the Lepr-biotin antibody (RnD Systems) was validated using *LeprCre::tdTomato* mice. **d)** Lepr+ cells per femur (n=4-5 mice/cohort) **e)** Expression of KitL and SDF1 in Lepr+ cells by qPCR (n=3 mice per cohort). **f)** Expression of Top 15 NF-kB signaling targets within Lepr+ cells. **g)** Osteoblast cells per femur (n=4-5 mice/cohort) **h)** Expression of KitL in osteoblast cells by qPCR (n=3 mice per cohort). **i)** Expression of Top 15 NF-kB signaling targets within osteoblast cells. Actb was used as reference gene for normalization. Error bars represent sample mean ± SEM. One-way ANOVA for multiple comparisons and Tukey's correction was performed to determine statistical significance. * $P \leq 0.05$; ** $P<0.01$; *** $P<0.001$; n.s. $P>0.05$.

Supplemental Figure 7

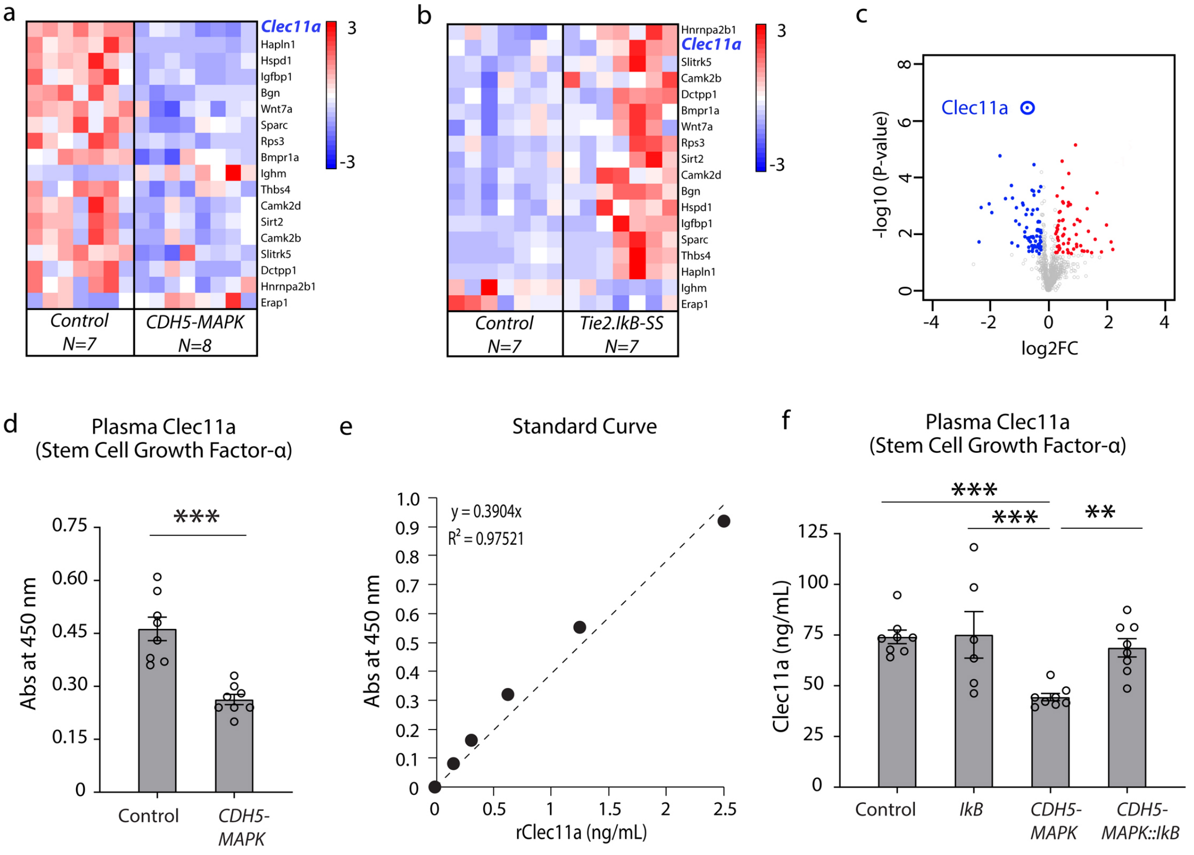

Supplemental Figure 7. Plasma proteome analysis identifies Clec11a/SCGF as a candidate pro-hematopoietic factor. Plasma was isolated from *CDH5-MAPK* and *Tie2.IkB-SS* mice, and their respective littermate controls. Proteomic analysis was performed by SomaLogic. 18 candidate factors were identified by analyzing proteins that showed significant differential expression (as compared to their littermate controls) and inverse correlation in *CDH5-MAPK* mice when compared to *Tie2.IkB-SS* mice. Heatmap displaying expression levels of candidate proteins in **a)** *CDH5-MAPK* mice and **b)** *Tie2.IkB-SS* mice. Note that *Clec11a* was significantly decreased in *CDH5-MAPK* mice and increased in *Tie2.IkB-SS* mice when compared to their controls. Raw data for Heatmaps included in Source Data. **c)** Volcano plot demonstrating that *Clec11a* was the most significant downregulated protein in *CDH5-MAPK* mice when compared to control mice. **d)** Aptamer based sandwich ELISA confirms decreased plasma Clec11a/SCGFα in *CDH5-MAPK* mice (n=8 mice/cohort). Error bars represent sample mean ± SEM. Statistical significance was determined using two-tailed unpaired Student's t-test. *** P<0.001. **e)** ELISA standard curve generated using recombinant SCGF to estimate plasma concentrations of *Clec11a*. **f)** Direct ELISA confirming that MAPK activation in endothelial cells results in decline of plasma *Clec11a* whereas inhibition of endothelial NF-κB signaling in *CDH5-MAPK* mice restores their plasma *Clec11a* to steady state levels (n=6-8 mice/cohort). Plasma was diluted 1:50 to ensure absorbance measurements were within the linear range of the assay. Error bars represent sample mean ± SEM. One-way ANOVA for multiple comparisons and Tukey's correction was performed to determine significance. * P≤0.05; ** P<0.01; *** P<0.001; n.s. P>0.05. Color scales represent relative protein expression.

Supplemental Figure 8

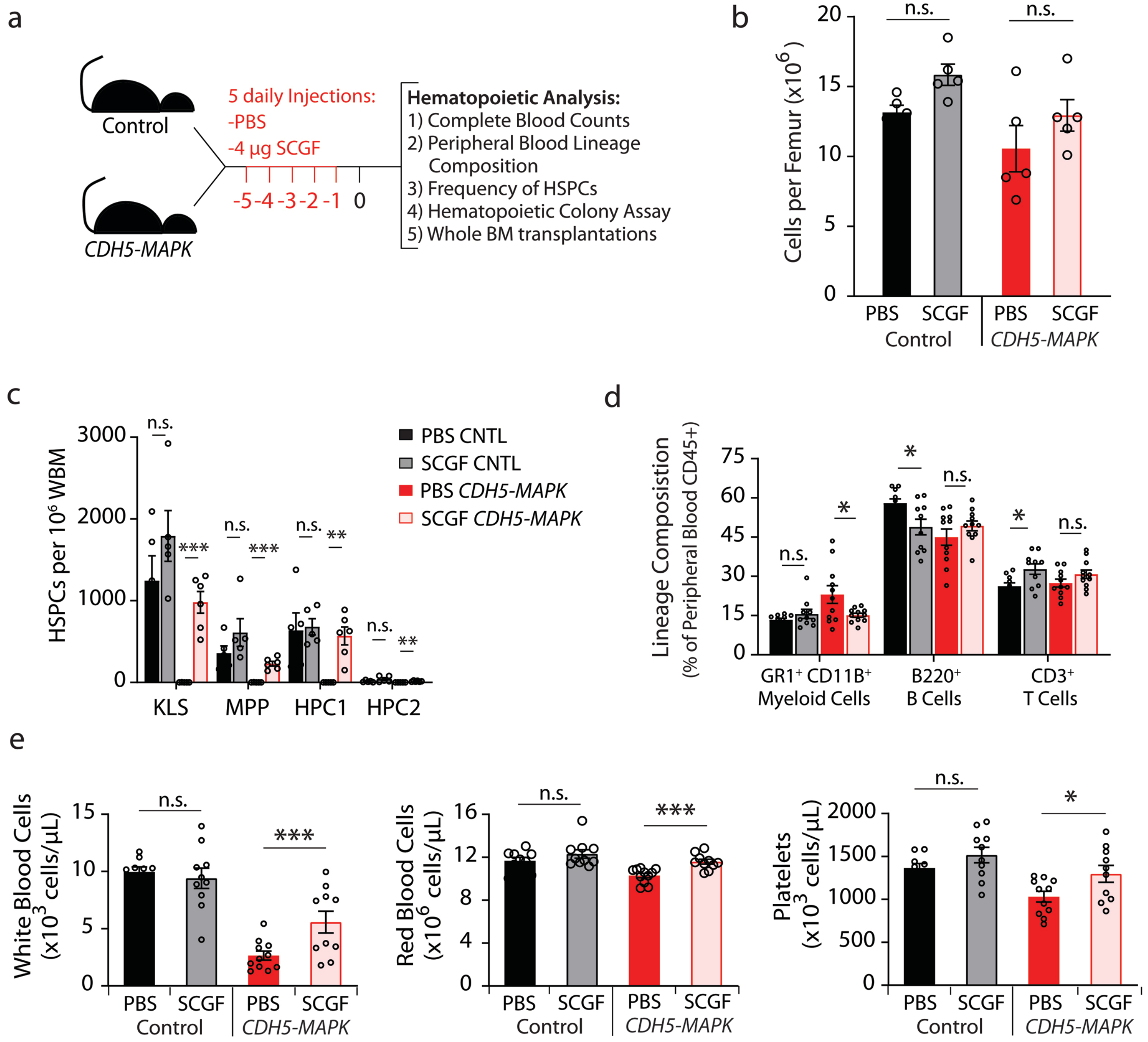

Supplemental Figure 8. SCGF infusion restores hematopoietic defects in *CDH5-MAPK* mice. a) Schematic diagram describing SCGF infusion regimen. 4 µg SCGF was injected subcutaneously every day for 5 consecutive days prior to analysis. **b)** Total cells per femur (n=5 mice/cohort). **c)** Frequency of phenotypic HSPCs per 10^6 femur cells assessed by flow cytometry (n=5-6 mice/cohort). **d)** Analysis of steady state peripheral blood lineage composition (n=10 mice/cohort). **e)** Complete blood counts demonstrating that SCGF treatment restored the levels of white blood cells, red blood cells, and platelets in *CDH5-MAPK* mice (n=9-11 mice/cohort). Error bars represent sample mean ± SEM. Statistical significance was determined using two-tailed unpaired Student's t-test. * P≤0.05; ** P<0.01; *** P<0.001; n.s. P>0.05.

Supplemental Figure 9

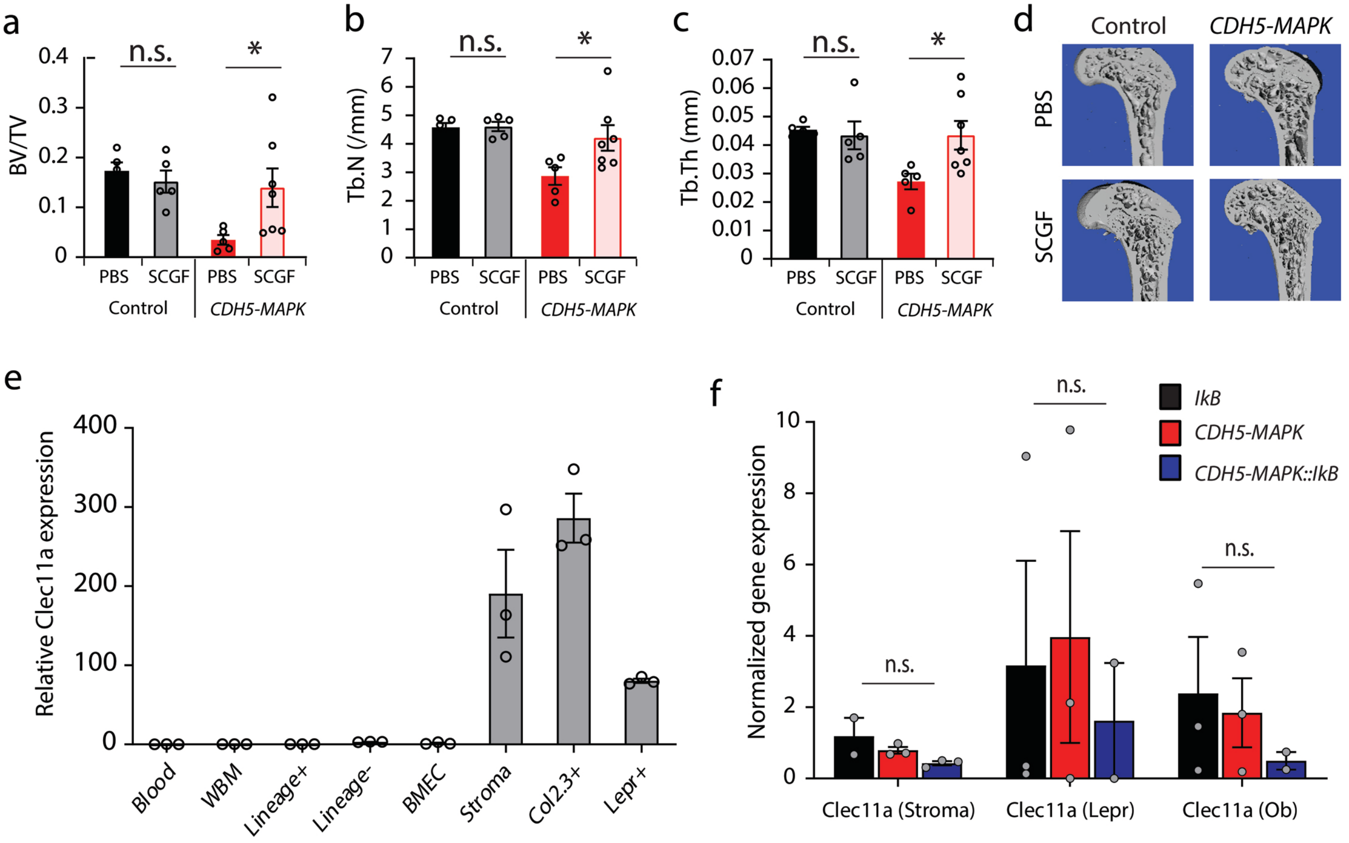

Supplemental Figure 9. SCGF infusion restores bone defects in *CDH5-MAPK* mice. a-d) Micro-computed tomography (µ-CT) analysis of trabecular bone revealed that *CDH5-MAPK* mice displayed a reduction in their **a)** bone volume/total volume (BV/TV), **b)** trabecular number (Tb.N) and **c)** trabecular thickness (Tb.Th) which show a significant improvement upon SCGF infusion (n=5-6 mice/cohort). **d)** Representative µ-CT images of femurs. **e)** Normalized gene expression of Clec11a/SCGF in the indicated cell types by RT-qPCR**.** Actb was used as reference gene for normalization (n=3 mice/cohort). Note that Clec11a is predominantly expressed in BM stromal cells, Lepr+ cells and osteoblasts. **f)** Normalized gene expression of Clec11a/SCGF in BM stromal cells, Lepr+ cells and osteoblasts in the indicated genotypes by RT-qPCR**.** *Actb* was used as reference gene for normalization (n=3 mice/cohort). Error bars represent sample mean ± SEM. Statistical significance was determined using two-tailed unpaired Student's t-test. * $P \leq 0.05$; ** $P<0.01$; *** $P<0.001$; n.s. $P>0.05$.

Supplemental Figure 10

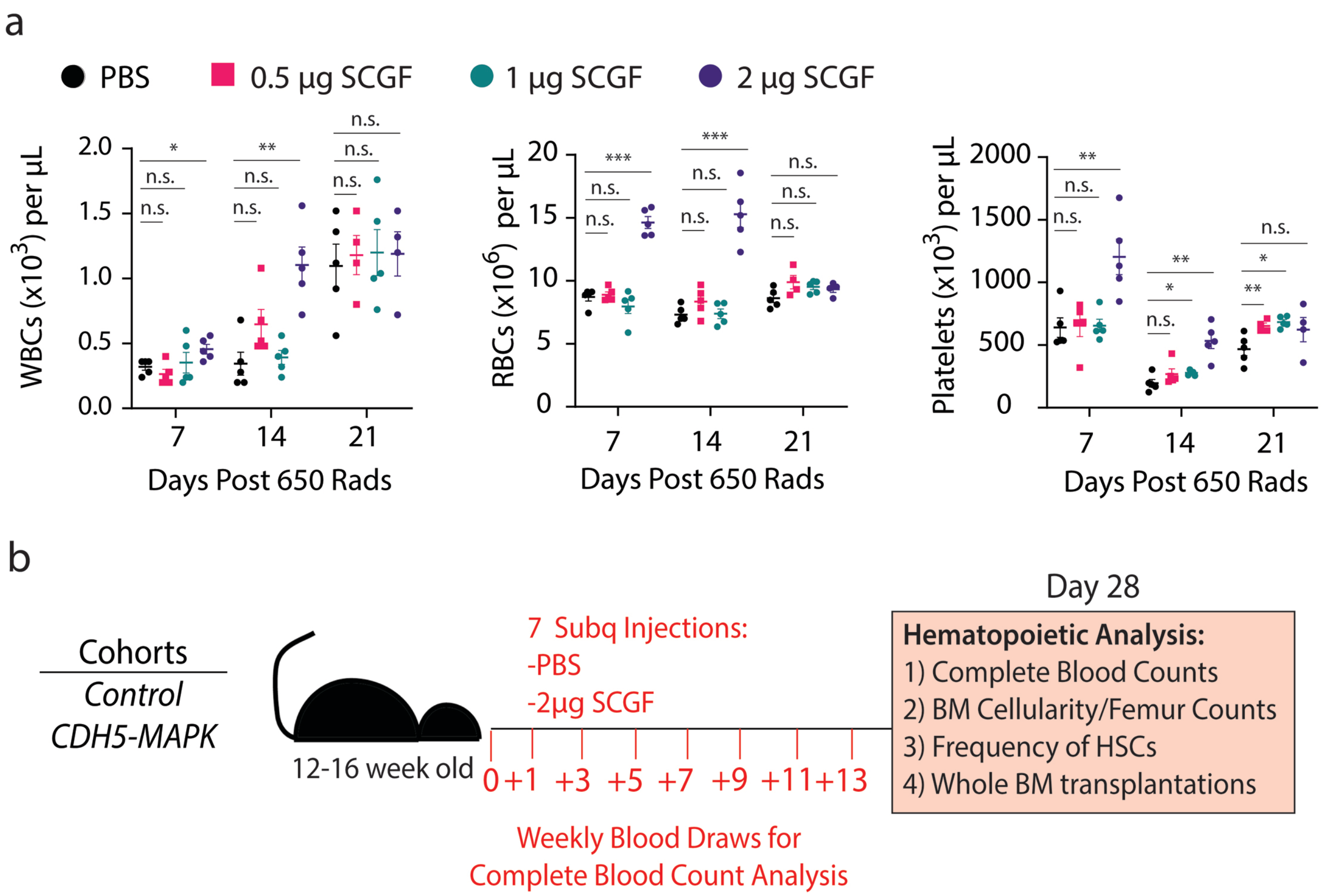

Supplemental Figure 10. Dose response of SCGF in promoting hematopoietic regeneration. a) Alternate day injections of 2 µg SCGF promotes earlier recovery of WBC, RBC and platelet counts following myelosuppressive irradiation (650 Rad). Error bars represent sample mean ± SEM (n=4-5 mice/cohort). Statistical significance was determined using two-tailed unpaired Student's t-test. * $P \leq 0.05$; ** $P<0.01$; *** $P<0.001$; n.s. $P>0.05$. **b)** Experimental scheme describing dosing regimen of SCGFα for assessment of hematopoietic regeneration.

Supplemental Table 1. List of proteins displayed in Heatmaps of Figure 2.

List of 97 proteins significantly upregulated in plasma of CDH5-MAPK mice as compared to control mice identified by Somalogics assay (n=7 Controls, n=8 CDH5-MAPK). (Related to Heatmap in Fig 2e).

Target	p-value	Log2 FC	Target	p-value	Log2 FC
MAPK5	7.07E-06	0.950	ALCAM	0.026	0.349
sICAM-1	2.64E-05	0.476	IL-1b	0.027	0.244
ST4S6	7.21E-05	0.677	CAMK1	0.028	0.310
SEPR	2.32E-04	0.497	41	0.028	1.004
Troponin I	3.52E-04	1.789	BGH3	0.032	0.403
VCAM-1	4.50E-04	0.296	DEAD-box protein 19B	0.033	0.430
IL-13 Ra1	7.25E-04	0.504	IL-17 RC	0.033	0.111
PTN	7.67E-04	0.724	IMDH1	0.034	0.906
GOT1	8.03E-04	0.534	SRCN1	0.035	2.443
Spondin-1	9.08E-04	0.759	M2-PK	0.036	0.359
IL-18 BPa	1.01E-03	0.683	RAC1	0.036	0.717
PYY	1.28E-03	1.432	GIB	0.039	0.178
VEGF	1.41E-03	0.227	LYNB	0.039	1.251
contactin-1	1.60E-03	0.398	CSK	0.041	1.409
Cadherin-2	1.71E-03	0.368	PDE5A	0.041	1.430
AMHR2	3.09E-03	1.038	SHP-2	0.043	2.073
WFKN2	3.16E-03	0.508	UFC1	0.044	0.544
PTP-1B	3.34E-03	0.481	NMT1	0.044	0.333
ROBO2	3.43E-03	0.368	NCC27	0.045	0.607
MIS	3.53E-03	1.200	IGFBP-5	0.045	0.256
DR3	4.66E-03	0.924	LYN	0.046	1.564
Cytochrome P450 3A4	4.82E-03	2.186	MK01	0.046	0.756
PKC-A	5.00E-03	2.545	PA2G4	0.049	0.910
MASP3	5.00E-03	0.335	LGMN	0.049	0.219
IL-13	5.20E-03	0.232	HIPK3	0.050	0.121
DLL1	6.11E-03	0.302	LCK	0.055	0.155
Angiogenin	6.52E-03	0.555	KPCT	0.056	1.303
Arylsulfatase A	6.83E-03	0.330	FGFR-2	0.057	1.109
Desmoglein-2	8.04E-03	1.382	ITI heavy chain H4	0.058	0.313
Rb	8.24E-03	0.287	MAPK14	0.060	1.152
DERM	9.80E-03	0.259	Elastase	0.060	0.179
IgM	1.04E-02	0.569	FSTL3	0.062	0.269
PKC-B-II	1.04E-02	1.018	Cyclophilin F	0.062	1.824
RTN4	1.15E-02	0.504	Calpastatin	0.063	0.215
IL-1 sR9	1.29E-02	0.090	ILT-2	0.064	0.433
Endostatin	1.30E-02	0.321	TWEAK	0.065	0.103
ETHE1	1.43E-02	0.311	CRTAM	0.065	0.594
XPNPEP1	1.54E-02	1.842	phosphoglycerate kinase 1	0.065	0.802
Cofilin-1	1.55E-02	0.777	EphA1	0.066	0.182
EPI	1.59E-02	0.155	NCK1	0.067	0.458
ADAM 9	1.63E-02	0.153	Ficolin-3	0.068	2.783
IR	1.70E-02	0.360	TXD12	0.069	0.188
IL-19	1.86E-02	0.231	DPP2	0.070	0.198
BTK	1.92E-02	2.208	EphA5	0.070	0.255
B7-H2	2.11E-02	0.750	FGFR4	0.071	0.178
SDF-1	2.22E-02	0.506	IFN-g R1	0.074	0.426
BARK1	2.33E-02	1.120	NPS-PLA2	0.074	0.122
Lysozyme	2.60E-02	1.183	MP2K2	0.074	0.337
NovH	2.63E-02	0.211			

List of 145 proteins significantly down-regulated in plasma of CDH5-MAPK mice. (Related to Heatmap in Fig 2e).

Target	p-value	Log2 Fold
SCGF-alpha	3.38E-07	-0.722
HPLN1	1.71E-05	-1.702
GDF-11	3.51E-05	-0.496
Lymphotactin	6.45E-05	-0.245
HSP 60	1.92E-04	-1.298
CNTF	2.11E-04	-0.274
Lymphotoxin a2/b1	2.23E-04	-0.164
iC3b	2.84E-04	-0.565
Karyopherin-a2	3.06E-04	-0.533
Aminoacylase-1	3.09E-04	-0.322
PSA2	4.23E-04	-0.492
CK-MB	5.35E-04	-1.284
IGFBP-1	5.62E-04	-1.506
Fucosyltransferase 3	6.82E-04	-0.148
HMG-1	8.12E-04	-0.855
IL-17D	8.23E-04	-0.228
FABP	8.58E-04	-2.053
BGN	9.36E-04	-0.906
Myokinase, human	1.15E-03	-2.202
b-ECGF	1.18E-03	-1.054
WNT7A	1.33E-03	-0.367
MDHC	1.36E-03	-0.725
PCNA	1.42E-03	-0.574
CK-MM	1.75E-03	-2.040
COX-2	2.00E-03	-0.552
ATP synthase beta chain	2.05E-03	-0.831
MLR1, isoform CRA_b	2.06E-03	-0.243
Granzyme B	2.07E-03	-0.197
CSK21	2.16E-03	-0.207
MK12	3.46E-03	-1.164
TLR2	3.60E-03	-0.354
NID2	3.70E-03	-0.308
ON	3.75E-03	-0.362
RS3	4.57E-03	-1.108
IL-15 Ra	4.65E-03	-0.187
FCN2	4.67E-03	-0.163
GIIE	5.17E-03	-0.155
ACTH	5.92E-03	-0.786
BMPR1A	7.04E-03	-0.362
Testican-2	7.09E-03	-0.324
MMP-10	7.32E-03	-0.200
TrATPase	7.46E-03	-0.280
b-Endorphin	7.56E-03	-0.407
Thrombin	7.70E-03	-0.374
Cyclin B1	7.90E-03	-0.216
MK13	8.02E-03	-0.227
Livin B	8.19E-03	-0.295
HSP 70	8.45E-03	-0.889
BSSP4	8.96E-03	-0.247

Target	p-value	Log2 FC
TGF-b3	0.009	-0.250
GAS1	0.009	-0.101
HCK	0.010	-0.250
TSP4	0.012	-0.401
TRY3	0.012	-0.248
SP-D	0.012	-0.524
Histone H2A.z	0.012	-0.252
CAMK2D	0.012	-0.845
PAPP-A	0.013	-0.163
Nidogen	0.013	-0.286
KPCI	0.013	-0.622
PLXC1	0.013	-0.114
SIRT2	0.014	-0.548
TS	0.014	-0.646
Glypican 3	0.014	-0.250
CAMK2B	0.014	-0.738
CBX5	0.015	-0.244
GM-CSF	0.015	-0.147
FSH	0.017	-0.663
TBP	0.017	-0.273
HVEM	0.017	-0.124
Apo D	0.017	-0.202
VEGF121	0.018	-0.466
Ku70	0.019	-0.258
CLC1B	0.019	-0.163
RBM39	0.019	-0.631
Myoglobin	0.019	-2.057
LIGHT	0.020	-0.159
MEPE	0.020	-0.145
GAPDH, liver	0.021	-1.543
BASI	0.022	-0.148
ENTP5	0.022	-0.333
IL-17E	0.024	-0.166
SLIK5	0.024	-0.331
PKC-D	0.024	-0.119
RELT	0.024	-0.229
IL-18 Ra	0.025	-0.145
GREM1	0.026	-0.133
STRATIFIN	0.026	-0.567
IL-8	0.026	-0.113
CAMK2A	0.026	-0.595
XTP3A	0.026	-0.500
Carbonic anhydrase III	0.027	-1.100
CD30	0.027	-0.167
KI2L4	0.027	-0.587
IF4A3	0.027	-0.386
PFD5	0.028	-0.362
AMNLS	0.028	-0.254
LKHA4	0.029	-0.113

List of 145 proteins significantly down-regulated in plasma of CDH5-MAPK mice. (Related to Heatmap in Fig 2e). (Continued from previous page)

Target	p-value	Log2 Fold
NANOG	2.87E-02	-0.312
p27Kip1	2.89E-02	-0.455
RPS6KA3	2.95E-02	-0.726
ANGL3	3.02E-02	-0.319
hnRNP A/B	3.06E-02	-0.579
Granzyme H	3.12E-02	-0.204
PSA	3.13E-02	-0.236
Nucleoside diphosphate kinase A	3.16E-02	-0.301
IL-34	3.18E-02	-0.235
IGF-II receptor	3.21E-02	-0.250
IL-1 sRI	3.24E-02	-0.177
Transketolase	3.27E-02	-0.568
AN32B	3.35E-02	-0.149
Thrombopoietin Receptor	3.47E-02	-0.154
MATN3	3.50E-02	-0.136
HPV E7 Type 16	3.60E-02	-0.132
BPI	3.64E-02	-0.280
BDNF	3.69E-02	-0.124
Triosephosphate isomerase	3.98E-02	-0.640
hnRNP A2/B1	4.03E-02	-0.415
Thymidine kinase	4.04E-02	-0.152
FGF23	4.10E-02	-0.715
ERK-1	4.11E-02	-0.601
IL-7	4.31E-02	-0.264
HAI-1	4.84E-02	-0.134
ANP	4.92E-02	-0.363
Protein disulfide-isomerase	5.04E-02	-0.145
PIM1	5.33E-02	-0.131
AURKB	5.40E-02	-0.123
MIP-1a	5.42E-02	-0.244
FABPE	5.48E-02	-1.102
CLC4K	5.50E-02	-0.175
C6	5.77E-02	-0.111
ATS15	5.84E-02	-0.101
TGF-b2	5.90E-02	-0.135
CAPG	6.13E-02	-0.579
ASGR1	6.23E-02	-0.087
C1r	6.25E-02	-0.247
RUXF	6.43E-02	-0.329
prostatic binding protein	6.53E-02	-0.355
Layilin	6.83E-02	-0.100
ALT	7.04E-02	-0.147
MFGM	7.23E-02	-0.204
cGMP-stimulated PDE	7.32E-02	-0.136
HSP 90a/b	7.34E-02	-0.187
CHST2	7.35E-02	-0.117
RANK	7.44E-02	-0.288

Supplemental Table 2. List of genes displayed in Heatmaps of Fig 3 and Supp. Fig. 4.

Normalized gene expression (average fold change) of NF-κB targets in BMECs. (Related to Heatmap in Fig 3e). * depict genes which are significantly upregulated ($p<0.05$) in CDH5-MAPK mice as compared to controls.

Gene ID	Control (n=3)	Cdh5-MAPK (n=3)	Cdh5-MAPK ::Tie2.IkBss (n=3)
Il1b	1.61	10.88	0.57
Cxcl3	2.46	7.27	0.79
Il2ra	2.48	6.50	26.45
Csf2	1.28	6.07	1.62
C3	1.23	5.65	0.03
Il2	1.64	4.02	1.21
Csf3	1.11	3.42	0.48
Snap25	1.64	2.69	0.09
Il1a	3.82	2.56	3.54
C4a	2.83	2.39	0.22
Mitf	1.02	2.27	3.37
Ccnd1*	1.02	2.26	1.25
Csf1	1.01	2.22	2.32
Plau*	1.06	2.03	1.44
Cd83	1.45	1.77	1.08
Il6	1.10	1.67	0.28
Cd80	2.38	1.61	0.14
Ifnb1	1.32	1.55	0.59
Cdkn1a	1.03	1.50	0.56
Ccl12	1.02	1.40	0.12
Map2k6	1.19	1.39	1.54
Nfkb1	1.02	1.37	0.92
Lta	3.04	1.35	0.14
Traf2	1.01	1.29	1.70
Ccl5	1.19	1.27	1.20
Icam1	1.00	1.18	0.50
Stat1	1.06	1.17	0.62
Egfr	1.41	1.11	0.45
Trp53	1.02	1.07	0.84
Xiap	1.00	1.07	0.67
Nfkb2	1.02	1.06	0.16
Akt1	1.03	1.05	0.80
Il1rn	1.78	1.03	1.21
Nr4a2	1.03	1.02	0.34
Cd40	1.48	1.00	2.24
Cxcl1	1.02	0.95	0.12
Bcl2l1	1.07	0.92	0.95
Il4	1.41	0.91	1.11
Mmp9	1.12	0.90	0.85
Stat3	1.01	0.89	0.42
Ccr5	4.05	0.89	0.13
Relb	1.00	0.88	0.67

Gene ID	Control (n=3)	Cdh5-MAPK (n=3)	Cdh5-MAPK ::Tie2.IkBss (n=3)
Rel	1.01	0.82	0.19
Rela	1.00	0.78	0.51
Pdgfb	1.03	0.77	1.26
Myc	1.00	0.76	0.41
Agt	1.37	0.76	3.54
Nfkbia	1.01	0.75	0.36
Tnfrsf1b	1.02	0.75	0.92
Fasl	1.14	0.75	0.51
Il1r2	1.84	0.74	1.88
Bcl2a1a	1.38	0.73	0.71
Tnfsf10	1.01	0.72	1.00
Csf2rb	1.02	0.71	0.91
Cfb	1.03	0.70	0.39
Egr2	1.02	0.70	0.58
Ifng	1.20	0.70	0.62
Sod2	1.03	0.68	0.58
F8	1.00	0.68	0.65
Aldh3a2	1.02	0.66	0.52
Stat5b	1.05	0.65	0.55
Selp	1.00	0.64	0.59
Cd74	1.52	0.61	0.21
Birc2	1.01	0.61	0.41
Vcam1	1.01	0.61	0.69
Fas	1.16	0.60	0.41
Il15	1.00	0.59	0.58
Cxcl9	1.04	0.58	0.14
Ncoa3	1.00	0.58	0.89
Il12b	1.21	0.56	6.41
Ins2	1.54	0.56	1.11
Birc3	1.00	0.55	0.29
Nqo1	1.58	0.53	0.77
Gadd45b	1.02	0.50	0.30
Adm	1.05	0.47	0.34
Sele	1.01	0.47	0.53
Myd88	1.01	0.41	0.34
Irf1	1.01	0.38	0.15
Ptgs2	1.89	0.33	0.09
Cxcl10	1.04	0.31	0.05
F3	3.02	0.30	0.34
Ccl22	2.78	0.22	0.12
Tnf	1.80	0.13	0.04
Ltb	1.12	0.06	1.97

Normalized gene expression (average fold change) of NF-κB targets in BM CD45+ cells. (Related to Heatmap in Supp. Fig 4a). * depict significantly upregulated genes ($p<0.05$) in CDH5-MAPK mice compared to controls.

**Gene ID**	**Control (n=3)**	**Cdh5-MAPK (n=3)**	**Cdh5-MAPK ::Tie2.IkBss (n=3)**
Il1a	2.33	10.11	0.72
Cxcl1	1.28	9.68	1.05
Ccl22	1.00	9.65	10.44
Il2ra*	1.16	7.81	1.20
Il2	1.00	7.80	1.81
Il1b*	1.02	6.47	0.42
Il4	2.60	6.02	3.20
Cdkn1a*	1.00	5.32	0.33
Ptgs2*	1.03	4.90	0.29
Csf3	1.00	4.80	1.81
Lta	4.04	4.59	2.94
Csf1*	1.05	4.44	0.99
Il12b	1.32	4.33	1.02
Cd80*	1.02	4.30	0.55
Cxcl3*	1.07	4.03	0.12
Ccl12	1.00	3.89	1.81
Bcl2a1a*	1.03	2.74	0.65
Egr2*	1.14	2.63	0.16
Gadd45b	1.00	2.61	2.71
Nqo1	1.00	2.46	1.98
Bcl2l1	1.01	2.39	1.03
Il1r2*	1.00	2.35	0.73
Il1rn*	1.03	2.32	1.06
Fas*	1.00	2.31	1.18
Sele	1.11	2.28	1.42
Il15*	1.01	2.21	1.24
Tnf*	1.00	2.19	1.17
Irf1	1.01	2.13	0.91
Csf2	1.01	2.06	0.84
Cxcl10*	1.06	2.05	0.26
Nfkbia*	1.00	1.89	1.12
Csf2rb*	1.00	1.70	1.01
Ifng	1.11	1.64	0.18
Snap25	1.32	1.55	2.11
Mmp9	1.00	1.47	1.18
Vcam1	1.04	1.47	1.06
Birc3	1.00	1.46	0.95
Myd88	1.01	1.45	0.84
Ccl5	1.02	1.44	0.56
Stat3	1.01	1.44	0.97
Stat1	1.01	1.44	0.84
C4a	2.27	1.42	0.19

**Gene ID**	**Control (n=3)**	**Cdh5-MAPK (n=3)**	**Cdh5-MAPK ::Tie2.IkBss (n=3)**
Rela	1.00	1.42	0.79
Tnfrsf1b	1.00	1.39	0.70
Agt	1.00	1.38	1.81
Cxcl9	1.00	1.38	1.81
F8	1.00	1.38	1.81
Ifnb1	1.00	1.38	1.81
Ltb	1.02	1.36	1.09
Relb	1.08	1.31	0.96
Selp	1.00	1.23	0.78
Nfkb2	1.01	1.23	0.63
Tnfsf10	1.11	1.18	1.25
Icam1	1.05	1.13	0.40
Stat5b	1.00	1.08	0.97
F3	1.01	1.06	0.19
Birc2	1.00	1.04	0.68
Pdgfb	1.08	0.99	0.41
Akt1	1.00	0.93	0.99
Ins2	1.26	0.92	1.21
Ncoa3	1.02	0.90	0.85
Cfb	1.02	0.87	1.02
Aldh3a2	1.01	0.86	0.83
Mitf	1.00	0.82	0.80
Fasl	1.05	0.77	0.34
Ccnd1	1.07	0.77	0.68
C3	1.00	0.73	1.03
Rel	1.00	0.71	0.50
Nr4a2	1.13	0.64	0.07
Nfkb1	1.00	0.64	0.61
Plau	1.02	0.63	0.84
Sod2	1.01	0.60	0.78
Trp53	1.00	0.56	0.59
Traf2	1.00	0.55	0.69
Xiap	1.00	0.55	0.75
Il6	1.06	0.51	0.03
Myc	1.04	0.50	0.33
Ccr5	1.02	0.44	0.30
Adm	1.71	0.25	0.33
Map2k6	1.07	0.23	0.56
Cd74	1.02	0.19	0.49
Cd40	1.01	0.19	0.66
Cd83	1.06	0.11	0.22
Egfr	1.06	0.06	0.62

Normalized gene expression (average fold change) of NF-κB targets in BM stromal cells. (Related to Heatmap in Supp. Fig 4c). * depict significantly upregulated genes ($p<0.05$) in CDH5-MAPK mice compared to controls.

Gene ID	Control (n=3)	Cdh5-MAPK (n=3)	Cdh5-MAPK ::Tie2.IkBss (n=3)
Cd80	4.26	976.63	168.64
Fas	1.32	256.69	21.38
Cfb	1.23	221.95	110.08
Agt	10.16	183.67	0.20
Ptgs2	8.57	170.52	5.85
Il1b	2.94	138.60	5.74
Csf2rb	2.18	78.69	67.85
Cxcl9	159.34	64.65	0.02
Csf1	15.85	61.49	0.01
Ccl5	3.08	61.25	17.12
Ltb	6.98	53.54	122.33
Bcl2a1a	1.32	41.65	14.89
Icam1	18.40	34.59	535.99
Egfr	13.75	32.06	0.06
Cd83	1.17	19.90	3.21
Cdkn1a	1.10	16.78	0.36
Sele	1.29	15.22	0.72
Il15*	2.54	13.64	0.97
Il1rn*	2.03	11.94	0.94
Ccnd1	2.70	11.67	4.10
Lta	1.22	10.42	3.40
Cd40	1.08	7.32	5.12
Tnfrsf1b	2.56	6.44	1.47
Birc3	1.07	6.12	2.15
Gadd45b	1.20	5.71	1.74
Plau	1.37	5.54	1.66
Cxcl1	23.77	5.30	0.04
Aldh3a2	1.19	3.78	2.12
Stat3	1.77	3.70	1.59
Il1r2	1.14	3.51	0.67
Mmp9	1.11	3.49	2.23
Cd74	1.38	3.32	0.09
Cxcl3	1.36	2.89	370.82
Irf1	2.34	2.79	0.09
Pdgfb	1.38	2.67	1.88
Akt1	1.01	2.54	2.34
Il12b	1.00	2.50	1.41
Bcl2l1	2.32	2.25	0.67
Myd88	1.83	2.20	1.57
Nfkb1	1.08	2.16	3.00
Vcam1	1.93	2.15	0.29
Birc2	1.28	1.99	0.40

Gene ID	Control (n=3)	Cdh5-MAPK (n=3)	Cdh5-MAPK ::Tie2.IkBss (n=3)
Rel	1.55	1.94	0.34
Csf2	1.23	1.89	1.73
Ifnb1	1.35	1.68	1.17
Mitf	1.87	1.60	0.40
Tnf	1.25	1.36	0.67
Egr2	1.24	1.26	3.13
Nfkb2	1.55	1.14	0.89
Cxcl10	4.51	1.12	0.01
Ccl22	1.54	1.06	1.04
Stat1	1.01	1.06	1.36
C3	1.01	1.02	0.91
Nfkbia	1.05	0.98	0.40
C4a	1.08	0.97	0.77
Il2	1.00	0.83	0.80
Snap25	2.52	0.72	0.61
F8	1.25	0.67	0.45
Xiap	1.36	0.67	0.41
Traf2	1.01	0.66	0.30
Selp	1.19	0.66	0.33
Il4	1.50	0.62	0.71
Csf3	1.00	0.60	0.79
Il2ra	1.00	0.59	0.41
Tnfsf10	1.00	0.58	6.48
Ccl12	1.00	0.56	137.14
Fasl	1.00	0.56	0.41
Il6	1.00	0.54	1.23
Ncoa3	1.15	0.52	0.48
Rela	1.13	0.48	0.67
Relb	2.37	0.44	0.23
Stat5b	1.07	0.37	0.08
F3	3.87	0.36	0.08
Adm	2.31	0.35	0.49
Sod2	1.08	0.22	0.15
Ins2	3.37	0.19	0.31
Trp53	1.03	0.12	0.14
Ifng	10.19	0.09	0.07
Myc	1.10	0.08	0.02
Map2k6	2.44	0.05	0.14
Ccr5	73.14	0.04	0.03
Il1a	9.48	0.03	2.73
Nqo1	1.06	0.01	0.16
Nr4a2	30.67	0.01	0.01

Normalized gene expression (average fold change) of NF-κB targets in WBM cells. (Related to Heatmap in Supp. Fig 4e). * depict significantly upregulated genes ($p<0.05$) in CDH5-MAPK mice as compared to controls.

Gene ID	Control (n=3)	Cdh5-MAPK (n=3)	Cdh5-MAPK ::Tie2.IkBss (n=3)
Ccl12*	1.01	42.43	2.71
Ccl22	1.09	34.22	7.47
Cxcl9	1.01	31.06	17.07
Agt	3.31	25.47	0.62
Il12b	1.01	21.92	1.02
Ifng*	1.08	10.68	0.94
Fasl*	1.02	10.40	0.90
Ccl5*	1.01	8.14	1.48
Ccnd1*	1.01	8.06	1.39
Egfr*	1.05	8.00	1.89
Pdgfb*	1.11	7.60	1.18
C4a	2.26	7.16	2.13
Lta*	1.11	6.67	0.74
Il2	2.93	6.21	2.66
Ccr5*	1.01	5.69	1.36
Nr4a2	1.04	5.42	1.57
F8	1.01	5.29	0.60
Tnfsf10*	1.01	4.86	1.09
Bcl2a1a*	1.01	4.51	1.35
Cxcl3	2.01	4.06	2.18
Cdkn1a*	1.02	3.62	1.13
Egr2	1.17	3.59	0.67
Myc*	1.01	3.05	0.60
Il1b*	1.02	2.95	1.15
Trp53*	1.01	2.76	0.74
Sele	1.01	2.52	1.45
Ptgs2	1.04	2.44	0.63
Icam1*	1.00	2.41	1.11
Ltb*	1.01	2.39	1.09
Cd83*	1.00	2.37	0.83
Sod2*	1.01	2.37	0.75
Cd74*	1.01	2.33	0.69
Snap25	1.36	2.29	1.28
Il1a	1.16	2.12	0.94
Selp*	1.01	2.05	0.94
Csf1*	1.01	2.02	1.01
Ins2*	1.01	1.96	1.02
Map2k6*	1.00	1.94	1.10
Relb	1.00	1.93	1.17
Cd80*	1.00	1.90	1.44
Nfkb1*	1.00	1.90	1.12
Il2ra	1.01	1.85	1.49

Gene ID	Control (n=3)	Cdh5-MAPK (n=3)	Cdh5-MAPK ::Tie2.IkBss (n=3)
Birc2*	1.01	1.84	1.02
Irf1*	1.00	1.84	1.04
Traf2*	1.00	1.77	0.92
Nfkb2	1.05	1.75	1.63
Il6	1.10	1.69	0.77
Mitf	1.00	1.68	1.40
Bcl2l1	1.00	1.62	1.19
Stat1*	1.00	1.60	1.04
Ncoa3*	1.00	1.58	1.12
Akt1*	1.00	1.58	1.03
Stat5b*	1.00	1.56	0.99
Il1rn	1.00	1.51	1.21
Nqo1	1.04	1.48	0.58
Il1r2	1.00	1.45	1.07
Csf2rb	1.00	1.41	1.09
Xiap	1.00	1.39	1.13
Rela	1.00	1.38	0.92
Stat3	1.00	1.33	1.10
Gadd45b	1.00	1.25	0.92
Rel	1.00	1.22	1.22
Il15	1.01	1.17	1.29
Birc3	1.00	1.16	1.06
Tnfrsf1b	1.00	1.15	1.38
Fas	1.00	1.15	1.09
Cxcl10	1.01	1.14	1.10
Csf3	1.10	1.13	0.69
Myd88	1.00	1.10	1.06
Ifnb1	2.11	1.08	0.38
Csf2	1.02	1.08	0.71
Cfb	1.00	1.05	1.47
Vcam1	1.00	1.05	0.41
Cd40	1.01	0.95	1.38
Aldh3a2	1.00	0.94	1.10
Nfkbia	1.00	0.91	1.29
F3	1.08	0.87	1.36
Tnf	1.00	0.86	0.83
Mmp9	1.00	0.81	1.08
Cxcl1	2.90	0.72	0.37
Plau	1.01	0.66	1.31
Il4	1.03	0.63	0.39
C3	1.00	0.35	1.09
Adm	1.94	0.27	2.71

Supplemental Table 3. List of proteins differentially expressed in plasma of Tie2.IkBss mice.

List of 82 proteins differentially expressed in plasma of Tie2.IkBss mice as compared to control mice identified by SomaLogic assay (n=7 Controls, n=7 Tie2.IkBss).

Target	p-value	Log2 FC	Target	p-value	Log2 FC
HPLN1	4.30E-02	3.104	FN1.4	0.059	0.373
PTN	8.02E-05	1.780	Gro-a	0.071	0.361
TSP4	5.19E-02	1.529	JAG1	0.032	0.354
ON	2.62E-02	1.512	ROBO2	0.066	0.349
IGFBP-1	3.93E-02	1.492	EP15R	0.071	0.329
PKC-A	6.80E-02	1.410	HO-2	0.011	0.290
vWF	1.06E-04	1.210	TGF-b1	0.050	0.284
WIF-1	3.34E-02	1.206	HPG-	0.050	0.218
URB	3.34E-02	1.148	ERP29	0.064	0.217
HTRA2	3.33E-02	0.968	GAS1	0.057	0.211
CDK2/cyclin A	6.73E-03	0.962	Cathepsin D	0.056	0.144
Nidogen	1.63E-02	0.908	Coagulation Factor X	0.034	-0.191
MATN2	2.64E-02	0.885	Carbonic Anhydrase IV	0.036	-0.218
HSP 60	2.43E-02	0.866	IL-17 RD	0.049	-0.271
NID2	1.39E-02	0.849	Angiogenin	0.039	-0.343
BGN	2.89E-03	0.820	Ephrin-A4	0.070	-0.355
Semaphorin-6A	3.32E-02	0.820	GFRa-1	0.056	-0.362
CAMK2D	1.47E-02	0.814	B7-2	0.045	-0.373
PPase	4.04E-02	0.811	IL-7 Ra	0.074	-0.385
IDE	1.36E-02	0.767	KI3S1	0.013	-0.396
BOC	4.81E-02	0.752	BCL2-like 1 protein	0.023	-0.424
Glypican 3	1.91E-02	0.752	GREM1	0.031	-0.474
Stanniocalcin-1	2.78E-02	0.736	DcR3	0.051	-0.487
IL-22	2.14E-02	0.709	Somatostatin-28	0.043	-0.499
SIRT2	4.90E-02	0.693	Siglec-6	0.063	-0.517
41	2.93E-02	0.678	IgM	0.059	-0.573
NLGNX	1.42E-02	0.608	OMD	0.071	-0.574
BMPR1A	3.43E-02	0.568	Fucosyltransferase 3	0.042	-0.611
PFD5	4.48E-02	0.563	VEGF sR3	0.007	-0.640
XTP3A	5.10E-02	0.561	BST1	0.063	-0.649
Glucagon	3.82E-02	0.561	SLPI	0.033	-0.828
WNT7A	5.98E-02	0.559	DR3	0.017	-1.123
IGF-I sR	4.74E-02	0.545	Desmoglein-2	0.024	-1.510
SCGF-alpha	7.36E-02	0.523			
SLIK5	3.25E-02	0.516			
BGH3	4.95E-02	0.515			
Macrophage mannose receptor	1.44E-02	0.490			
CAMK2B	3.62E-02	0.490			
Flt-3	6.86E-02	0.488			
sE-Selectin	5.47E-02	0.462			
TSP2	6.65E-02	0.458			
WISP-1	1.25E-02	0.451			
Periostin	3.17E-02	0.450			
PIK3CA/PIK3R1	4.40E-02	0.441			
hnRNP A2/B1	4.03E-02	0.429			
YES	6.78E-02	0.429			
CD36 ANTIGEN	6.28E-02	0.401			
MMP-8	3.70E-02	0.395			
BMP-1	5.09E-02	0.380			

Supplemental Table 4. List of antibodies utilized for Flow Cytometry, ELISA and immunoblot analysis. All antibodies utilized at manufacturer recommended dilutions.

**Antibody**	**Vendor**	**Catalog**
Rat monoclonal anti-CD117 (2B8)	Biolegend	Cat# 105812
Rat monoclonal anti-Ly6A/E (D7)	Biolegend	Cat# 108108
Rat monoclonal anti-CD150 (TC15-12F12.2)	Biolegend	Cat# 115914
Armenian Hamster monoclonal anti-CD48 (HM48-1)	Biolegend	Cat# 103414
Rat monoclonal anti-Ly6G/Ly6C (RB6-8C5)	Biolegend	Cat# 108430
Rat monoclonal anti-CD11b (M1/70)	Biolegend	Cat# 101224
Rat monoclonal anti-CD45R/B220 (RA3-6B2)	Biolegend	Cat# 103227
Rat monoclonal anti-CD3 (17A2)	Biolegend	Cat# 100214
Rat monoclonal anti-CD41 (MWReg30)	Biolegend	Cat# 133912
Rat monoclonal anti-TER119 (TER-119)	Biolegend	Cat# 116232
Rat monoclonal anti-CD16/32 (93)	Biolegend	Cat# 101320
Rat monoclonal anti-TER119 (TER-119)	Biolegend	Cat# 116211
Mouse monoclonal anti-CD45.1 (A20)	Biolegend	Cat# 110706
Mouse monoclonal anti-CD45.2 (104)	Biolegend	Cat# 109830
Rat monoclonal anti-CD45R/B220 (RA3-6B2)	Biolegend	Cat# 103212
Rat monoclonal anti-CD3 (17A2)	Biolegend	Cat# 100228
Rat monoclonal anti-CD4 (GK1.5)	Biolegend	Cat# 100423
Rat monoclonal anti-CD8a (53-6.7)	Biolegend	Cat# 100708
Rat monoclonal anti-CD31 (MEC13.3)	Biolegend	Cat# 102512
Rat monoclonal anti-CD144 (BV13)	Biolegend	Cat# 138006
Rat monoclonal anti-CD31 (390)	Biolegend	Cat# 102418
Rat monoclonal anti-CD45 (30-F11)	Biolegend	Cat# 103122
Rat monoclonal anti-CD127/IL-7Rα (A7R34)	Biolegend	Cat# 135008
Rat Monoclonal anti-CD135/Flt3 (A2F10)	Thermo Fisher	Cat# 12-1351-82
Rat Monoclonal anti-CD16/32 (93)	Biolegend	Cat# 101308
Rat Monoclonal anti-CD34 (93)	Thermo Fisher	Cat# 11-0341-82
Rat Monoclonal anti-mouse/human B220	Biolegend	Cat# 103227
Rat Monoclonal anti-IgM	Thermo Fisher	Cat# 17-5790-82
Rat Monoclonal anti-CD43	BD Biosciences	Cat# 561857
Rat Monoclonal anti-CD24 (M1/69)	Biolegend	Cat# 101822
Goat Polyclonal anti-LepR, Biotin Conjugated	R and D Systems	Cat# BAF497
Streptavidin Brilliant Violet 421™	Biolegend	Cat# 405225
Streptavidin APC	Biolegend	Cat# 405207
Rat Monoclonal anti-CD51 (RMV7)	Biolegend	Cat# 104106
Mouse Monoclonal anti-Ki67 (B56)	BD Biosciences	Cat# 561284
Mouse Isotype Control (MOPC-21)	BD Biosciences	Cat# 550795
Mouse Monoclonal anti-hypoxyprobe	Hypoxyprobe Inc.	Cat# HP7-100
Rat monoclonal anti-CD127/IL-7Rα (A7R34)	Biolegend	Cat# 135008
Rat monoclonal anti-CD45 (30-F11)	Biolegend	Cat# 103126
Rat anti-mouse Lineage cocktail	Biolegend	Cat# 133310
Rat monoclonal anti-CD31 (MEC13.3)	Biolegend	Cat# 102506
Rat monoclonal anti-TER119 (TER119)	Biolegend	Cat# 116206
Rat monoclonal anti-CD45 (30F-11)	Biolegend	Cat# 103108
Rat monoclonal anti-Ly6A/E (D7)	Biolegend	Cat# 108114
Rat monoclonal anti-CD45 (30-F11)	Biolegend	Cat# 103114

Rat monoclonal anti-TER119 (TER119)	Biolegend	Cat# 116222
Rat monoclonal anti-TER119 (TER119)	Biolegend	Cat# 116206
Rat monoclonal anti-CD3 (17A2)	Biolegend	Cat# 100204
Rat monoclonal anti-CD45R/B220 (RA3-6B2)	Biolegend	Cat# 103206
Rat monoclonal anti-CD11b (M1/70)	Biolegend	Cat# 101206
Rat monoclonal anti-Ly6G/Ly6C (RB6-8C5)	Biolegend	Cat# 108406
Rat monoclonal anti-CD41 (MWReg30)	Biolegend	Cat# 133904
Rabbit monoclonal anti-phospho-p65 (93H1)	Cell Signaling	Cat# 3033
Rabbit monoclonal anti-p65 (C22B4)	Cell Signaling	Cat# 4764
Rabbit monoclonal anti-phospho-Erk1/2 (D13.14.4E)	Cell Signaling	Cat# 4370
Rabbit polyclonal anti-Erk1/2	Cell Signaling	Cat# 9102
Mouse monoclonal anti-IκBα	Cell Signaling	Cat# 4814
Rabbit polyclonal anti-betaTubulin	Cell Signaling	Cat# 2146
Donkey polyclonal anti-rabbit Horseradish Peroxidase	Jackson ImmunoRes.	Cat# 711-035-152
Donkey polyclonal anti-mouse Horseradish Peroxidase	Jackson ImmunoRes.	Cat# 711-035-150
Goat polyclonal anti-SCGF/Clec11a	R&D Systems	Cat# AF3729
Donkey polyclonal anti-goat Horseradish Peroxidase	Jackson ImmunoRes.	Cat# 705-035-147

Supplemental Table 5. List of antibodies utilized for whole mount immunofluorescence (Related to Figure 4).

**Antibody (stock)**	**Fluor**	**Clone**	**Dilution**	**Company**
Ter119 (1mg/mL)	Pac-Blue	Ter119	1:100	Biolegend
CD41 (1mg/mL)	Pac-Blue	MWReG30	1:100	Biolegend
CD11b (1mg/mL)	Pac-Blue	M1/70	1:100	Biolegend
Gr1 (1mg/mL)	Pac-Blue	RB6-8C5	1:100	Biolegend
B220 (1mg/mL)	Pac-Blue	RA3-6B2	1:100	Biolegend
CD3 (1mg/mL)	Pac-Blue	17A2	1:50	Biolegend
CD48 (1mg/mL)	Pac-Blue	HM48-1	1:100	Biolegend
CD150 (0.2 mg/mL)	PE/Dazzle 594	TC15-12F12.2	1:50	Biolegend
CD144 ((1mg/mL)	AF647	BV13	25 μg (intravital)	Biolegend

Supplemental Table 6. List of primers. (Related to Figures 6, 7 and Supplemental Figures 6, 9).

**Gene**	**Forward Primer (5'-3')**	**Reverse Primer (5'-3')**
Actb	TGGCACCACACCTTCTACAATGAGC	CCAGAGGCATACAGGGACAGCACAG
Ptprc	CAGGGTCCACCTACATAAATGCCA	CCTTCTTCACATCGTGTGACCATGAC
Cre	ATGTCCAATTTACTGACCGTACACCA	ACGATGAAGCATGTTTAGCTGGCCCA
Cdh5	GAGAGACTGGATTTGGAATCAAATGCAC	CTCATAGGCAAGCACATTCCCTGTG
IkBa.SS	AGACCTGGCTTTCCTCAACTTCC	CAGCACCCAAGGACACCAAAAGC
Vcam1	GTTCCAGCGAGGGTCTACC	AACTCTTGGCAAACATTAGGTGT
Sele	ATGCCTCGCGCTTTCTCTC	GTAGTCCCGCTGACAGTATGC
Kitl	GAATCTCCGAAGAGGCCAGAA	GCTGCAACAGGGGGTAACAT
Cxcl12	TGCATCAGTGACGGTAAACCA	TTCTTCAGCCGTGCAACAATC
Jag1	CCTCGGGTCAGTTTGAGCTG	CCTTGAGGCACACTTTGAAGTA
Clec11a	GTCTGCACGTTTTGGACACC	GCCTGGGTCTCGAAGTCTC
