# Supplementary material for: Chronic activation of endothelial MAPK disrupts hematopoiesis via NFKB dependent inflammatory stress reversible by SCGF
Source: Nat Commun. 2020 Feb 3;11:666. doi: 10.1038/s41467-020-14478-8 (PMC6997369; doi:10.1038/s41467-020-14478-8)
Supplement: Supplementary file 4 — Reporting Summary [file 41467_2020_14478_MOESM4_ESM.pdf]

nature research

Corresponding author(s): Jason M Butler

Last updated by author(s): Dec 20, 2019

Reporting Summary

Nature Research wishes to improve the reproducibility of the work that we publish. This form provides structure for consistency and transparency in reporting. For further information on Nature Research policies, see Authors & Referees and the Editorial Policy Checklist.

Statistics

For all statistical analyses, confirm that the following items are present in the figure legend, table legend, main text, or Methods section.

n/a	Confirmed	
☐	☒	The exact sample size (*n*) for each experimental group/condition, given as a discrete number and unit of measurement
☐	☒	A statement on whether measurements were taken from distinct samples or whether the same sample was measured repeatedly
☐	☒	The statistical test(s) used AND whether they are one- or two-sided<br>*Only common tests should be described solely by name; describe more complex techniques in the Methods section.*
☒	☐	A description of all covariates tested
☐	☒	A description of any assumptions or corrections, such as tests of normality and adjustment for multiple comparisons
☐	☒	A full description of the statistical parameters including central tendency (e.g. means) or other basic estimates (e.g. regression coefficient) AND variation (e.g. standard deviation) or associated estimates of uncertainty (e.g. confidence intervals)
☐	☒	For null hypothesis testing, the test statistic (e.g. *F*, *t*, *r*) with confidence intervals, effect sizes, degrees of freedom and *P* value noted<br>*Give P values as exact values whenever suitable.*
☒	☐	For Bayesian analysis, information on the choice of priors and Markov chain Monte Carlo settings
☒	☐	For hierarchical and complex designs, identification of the appropriate level for tests and full reporting of outcomes
☒	☐	Estimates of effect sizes (e.g. Cohen's *d*, Pearson's *r*), indicating how they were calculated

Our web collection on statistics for biologists contains articles on many of the points above.

Software and code

Policy information about availability of computer code

Data collection	No custom code utilized for data collection.
Data analysis	1. Extreme Limiting Dilution Analysis (ELDA) software (http://bioinf.wehi.edu.au/software/elda/).<br>2. SomaSuite Version 1.0.3 software (http://somalogic.com)<br>3. Ingenuity Pathway Analysis (IPA) software (https://www.qiagenbioinformatics.com/products/ingenuity-pathway-analysis/)<br>4. GeneGlobe Data Analysis Center software (https://www.qiagen.com/in/shop/genes-and-pathways/data-analysis-center-overview-page/)<br>5. Prism 6 Version 6.07 (Graphpad)<br>6. Venny 2.1 (http://bioinfogp.cnb.csic.es/tools/venny/)<br>7. Image J (Version 1.51j8)

For manuscripts utilizing custom algorithms or software that are central to the research but not yet described in published literature, software must be made available to editors/reviewers. We strongly encourage code deposition in a community repository (e.g. GitHub). See the Nature Research guidelines for submitting code & software for further information.

Data

Policy information about availability of data

All manuscripts must include a data availability statement. This statement should provide the following information, where applicable:

- Accession codes, unique identifiers, or web links for publicly available datasets
- A list of figures that have associated raw data
- A description of any restrictions on data availability

The authors declare that data supporting the findings of this study are available within the manuscript and its supplementary information files. Source data for Figures 1-9 are provided with the paper as a Source Data File. Source data for Supplementary Figures will be provided upon request. Proteomics Raw Data are included in the Source Data File.

Field-specific reporting

Please select the one below that is the best fit for your research. If you are not sure, read the appropriate sections before making your selection.

☒ Life sciences ☐ Behavioural & social sciences ☐ Ecological, evolutionary & environmental sciences

For a reference copy of the document with all sections, see nature.com/documents/nr-reporting-summary-flat.pdf

Life sciences study design

All studies must disclose on these points even when the disclosure is negative.

Sample size	Sample sizes for phenotypic and functional analysis of mouse hematopoietic parameters were determined based on prior estimates of variance and effect sizes observed in previous experiments. Number of animals needed were calculated based on the ability to detect a two-fold change in the Mean with 80% power, with the threshold for significance (α) set at 0.05.
Data exclusions	Outliers were removed based on pre-determined exclusion criterion of any value which falls outside of Group Mean +/- 3 Standard Deviations. Outliers were not excluded in groups with N<5 samples per cohort.
Replication	All experimental findings in mouse studies were confirmed in at least 2 independent cohorts of mice and were reproducible.
Randomization	Randomization was not used.
Blinding	Blinding was not used.

Reporting for specific materials, systems and methods

We require information from authors about some types of materials, experimental systems and methods used in many studies. Here, indicate whether each material, system or method listed is relevant to your study. If you are not sure if a list item applies to your research, read the appropriate section before selecting a response.

Materials & experimental systems

n/a	Involved in the study	
☐	☒	Antibodies
☐	☒	Eukaryotic cell lines
☒	☐	Palaeontology
☐	☒	Animals and other organisms
☒	☐	Human research participants
☒	☐	Clinical data

Methods

n/a	Involved in the study	
☒	☐	ChIP-seq
☐	☒	Flow cytometry
☒	☐	MRI-based neuroimaging

Antibodies

Antibodies used	All antibodies used in the study are Listed in Supplemental Table 4.
Validation	All antibodies are commercially validated.

Eukaryotic cell lines

Policy information about cell lines

Cell line source(s)	Derived from mouse bone marrow-derived endothelial cells.
Authentication	Endothelial identity validated by Flow cytometry, as described in the Methods.
Mycoplasma contamination	Not determined.
Commonly misidentified lines (See ICLAC register)	Not applicable.

Animals and other organisms

Policy information about studies involving animals; ARRIVE guidelines recommended for reporting animal research

Laboratory animals	C57BL/6J (CD45.2; stock no. 000664), B6.SJL-Ptprca Pepcb/BoyJ (CD45.1; stock no. 002014), and C57BL/6-Gt(ROSA)26Sortm(Map2k1*,EGFP)Rsky/J (Mapkfl/fl) (stock no. 012352) mice were purchased from The Jackson Laboratory (Bar Harbor, ME). Cdh5(PAC)-creERT2 mice were obtained from Ralf H. Adams at The Max Planck Institute for Molecular Biomedicine. Tie2.IkB-SS mice were obtained from Jan Kitajewski at Columbia University. Lepr-cre mice were obtained from Sean J Morrison at the University of Texas Southwestern Medical Center. Mapkfl/fl, Cdh5(PAC)-creERT2, and Tie2.IkB-SS mice were bred and maintained on a C57BL/6J (CD45.2) genetic background. All mice were housed in Positive Individual Ventilation (PIV) cages with HEPA-filtered air exchange (Thoren Caging Systems, Inc.) and maintained on Pico Lab Rodent Diet 20 (Lab Diet 5053) and water ad libitum. To induce Cdh5(PAC)-creERT2-mediated recombination, Tamoxifen (Sigma-Aldrich T5648) solubilized in Sunflower Oil (Sigma-Aldrich S5007) was administered via intraperitoneal injection (150 mg/kg body weight) at a dose of 30 mg/mL for three consecutive days at 8-12 weeks of age or fed Custom Teklad 2020 Feed supplemented with 0.025% w/w tamoxifen (Envigo) ad libitum at 6-10 weeks of age for four consecutive weeks. Age matched cre-negative littermate mice also underwent the same tamoxifen induction regimen and were utilized as controls. Both male and female mice were utilized for experiments. Mice were allowed to recover for 4 weeks post-tamoxifen induction prior to experimental analysis. All mice were maintained in specific-pathogen-free housing.
Wild animals	No wild animals utilized.
Field-collected samples	NA
Ethics oversight	All experiments were conducted in accordance with the Association for Assessment and Accreditation of Laboratory Animal Care, Intl. (AAALAC) and National Institutes of Health (NIH) Office of Laboratory Animal Welfare (OLAW) guidelines and under the protocols approved by Institutional Animal Care and Use Committee (IACUC) of the Center for Discovery and Innovation at Hackensack Meridian Health and Weill Cornell Medical College.

Note that full information on the approval of the study protocol must also be provided in the manuscript.

Flow Cytometry

Plots

Confirm that:

- [ ] The axis labels state the marker and fluorochrome used (e.g. CD4-FITC).
- [x] The axis scales are clearly visible. Include numbers along axes only for bottom left plot of group (a 'group' is an analysis of identical markers).
- [x] All plots are contour plots with outliers or pseudocolor plots.
- [ ] A numerical value for number of cells or percentage (with statistics) is provided.

Methodology

Sample preparation	Information included in the Methods section.
Instrument	Information included in the Methods section.
Software	Information included in the Methods section.
Cell population abundance	Information included in the Methods section and the Manuscript.
Gating strategy	Information included in the Methods section as well as the Figures and Supplemental Figures for Gating strategies for all poipulations utilized in the study.

- [x] Tick this box to confirm that a figure exemplifying the gating strategy is provided in the Supplementary Information.
